# Breeding Efficiency for Salt Tolerance in Alfalfa

**DOI:** 10.3390/life13112188

**Published:** 2023-11-10

**Authors:** Michael D. Peel, M. Rokebul Anower, Yajun Wu

**Affiliations:** 1 Forage and Range Research, Logan, UT 84322, USA; 2 NYS Department of Health, Albany, NY 12237, USA; 3 Department of Biology and Microbiology, South Dakota State University, Brookings, SD 57007, USA

**Keywords:** forage mass, breeding method, growth response, stem growth, crude protein, NDF

## Abstract

Alfalfa (*Medicago sativa* L.), one of the most extensively grown forage crops, is sensitive to saline soils. We measured the breeding efficiency for increased salt tolerance in alfalfa by comparing lines selected from BC79S, CS, and SII populations with their unselected parental means for forage mass and associated changes in stem length, leaf-to-stem ratio (LSR), number of nodes per stem, crude protein (CP) content, and neutral detergent fiber (NDF) content. The overall forage mass in the non-salt-stressed test (9562 kg ha^−1^) was greater (*p* < 0.001) than under salt stress (5783 kg ha^−1^), with a 40% production advantage. In the non-salt-stressed test, the BC79S and CS lines averaged at a 4% lower production than their parents, while SII lines had on average a 9% greater production. Conversely, in the salt-stressed test, all lines showed a 20% overall greater seasonal production than their parents. Some selected lines produced more forage mass in both the non-stressed and salt-stressed tests than their parents. The stem length, LSR, node number, CP content, and NDF content of the selected lines varied with respect to non-stressed vs. stressed, but they tended not to differ greatly from their respective parental means under either non- or salt-stressed conditions. The selection protocol provided a universal increase in forage mass under salt-stressed field conditions of the selected lines. Furthermore, we identified lines with forage mass values greater than their parental means under non- and salt-stressed field conditions.

## 1. Introduction

Alfalfa (*Medicago sativa* L.) is one of the most extensively grown forage crops with 32–40 million hectare under cultivation globally [1,2,3]. It has immense agronomic importance because of its high nutritional value, perennial growth, and nitrogen-fixing capabilities [4,5]. Unfortunately, many alfalfa-growing regions are affected by salinity, and alfalfa is relatively sensitive to this stress compared to other species [6]. Salt stress has a universal effect of reducing the growth of both shoots and leaves in particular, with growth rates being reduced over time [7]. It is reported that alfalfa production is impacted when soil salinity is above a threshold of 2.0 dS m^−1^ (~22 mM NaCl), with a 7.3% reduction for each dS m^−1^ (~11 mM NaCl) increase above the threshold [8,9]. Arid and semi-arid regions are particularly plagued by saline soils where wicking action draws salts to the soil surface, resulting in salinity concentrations that limit or prevent crop production [10,11].

Salt tolerance in crop plants has overall been associated with differences in morphological and physiological traits such as changes in the shoot and root growth, leaf-cuticle thickness, stomatal regulation, photosynthesis rate, and seed germination [12,13,14]. The chlorophyll content index, an indication of senescence or cell damage under salinity stress [15,16], has been reported to be higher in the leaves of alfalfa ecotypes with better salt tolerance [16,17]. Salt-tolerant lines of wheat display smaller, thicker leaves, resulting in a higher chloroplast density per unit of leaf area [7,18]. Similarly, Anower et al. [12,19] demonstrated a substantial increase in chlorophyll content, root and shoot dry weight, and soluble sugars, but a reduction in stomatal conductance, under saline conditions in alfalfa lines selected for salt tolerance.

The literature contains multiple reports of variability for salinity tolerance in many crops including alfalfa [20]. Even so, the development of highly salt-tolerant cultivars has been challenging [21]. The selection of salt-tolerant plants from saline fields or plots seems a logical step for most plant breeders; however, this procedure has not produced consistent results because soil salinity varies with time, location, soil type, and depth [22,23,24]. Smith [25] identified germination, seedling growth, and mature plant growth as stages at which alfalfa plants may be affected by salinity. Since selection for tolerance at germination can be accomplished in vitro, many of the efforts to improve alfalfa for salinity tolerance have focused on germination. As such, the literature is replete with examples of the evaluation of and selection for salt tolerance in alfalfa at germination [26,27,28,29,30,31], as well as for other crops [32,33]. However, little correlation appears to exist between tolerance at germination and later growth stages in crops including alfalfa [26,34,35]. 

The difficulty of effective selection for salt tolerance in this field highlights the need for a tool, or procedure, that minimizes uncontrolled variation. To be deemed successful, the tool must provide a measurable response to selection such as that described by Ceccarelli [36], where the selection response shows gains relative to a referenced variety. Peel et al. [37] developed a repeatable method for screening plants grown in silica sand medium for their relative ability to withstand increasing levels of salt. This method, used in a greenhouse breeding and screening project, led to the development of three populations with improved survival under saline conditions. Lines from this effort have been shown under salt stress to exhibit one or more of the following: greater root growth, reduced stomatal conductance, maintenance of relative water content by accumulating more soluble sugars, lower accumulation of Na^+^, and maintenance of ion homeostasis [12,19]. With these encouraging results, we can further test Ceccarelli’s [36] measure of breeding efficiency under field conditions. Furthermore, with the documented reductions in plant height or stem length, as well as leaf characteristics [7,38,39] associated with salt stress, it is important to document any changes in these characteristics, as well as changes to the forage nutritive value that have not been reported. Therefore, the objective of this study was (1) to measure the breeding efficiency of the selection for salt tolerance in alfalfa by comparing selected lines with their respective parents for forage mass under salt-stressed and non-salt-stressed field conditions, and (2) to further characterize the selected lines for changes in leaf-to-stem ratio, stem length, number of nodes per stem, and forage nutritive value in terms of their crude protein (CP) and neutral detergent fibers (NDFs).

## 2. Materials and Methods

### 2.1. Plant Materials

**Figure 1:**
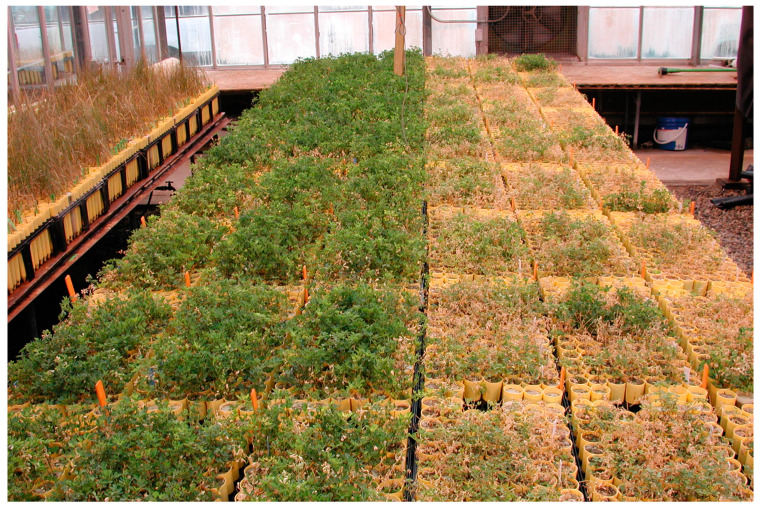
Alfalfa plants after 14 weeks of salt treatment following the greenhouse protocol of Peel et al. [37] for screening for salt tolerance. The plants on the right show little to no tolerance, while those on the left have been through two cycles of recurrent selection for their ability to survive high-salt conditions. At the time of the picture, the plants were subjected to an EC of 18 ds m^−1^ at a sodium adsorption ratio of 3.5.

Three alfalfa populations, BC79S, CS, and SII, were developed simultaneously using the method described by Peel et al. [37] following three cycles of recurrent selection for survival under increasing levels of salt (NaCl). Briefly, the seeds were germinated on blotter paper wetted with a salt solution (EC 9.0 ds m^−1^). The first seeds to germinate were transplanted into Ray Leach Cone-tainers (Stewe and Sons, Corvalis, OR, USA) and filled with 70-grit silica sand. After six weeks of growth, the seedlings were subjected to salt (NaCl) stress starting at an EC of 3 ds m^−1^, which was increased by 3 ds m^−1^ weekly until an EC of 18 ds m^−1^ was reached. All salt solutions were adjusted to a sodium-adsorption ratio of 3.5 with the addition of CaCl_2_. The salt concentration was maintained at an EC of 18 ds m^−1^ until the desired phenotypic separation of the plants was achieved, with approximately 98% mortality. Plants were watered twice weekly by submerging the entire 96-cone flat into a tank containing a nutrient solution with the desired level of salinity, flushing the silica sand, and bringing it into equilibrium with the desired salt concentration. Figure 1 shows the alfalfa plants after 14 weeks of salt treatment, comparing plants that have been through two cycles of selection next to unselected plants. Selected survivor plants were rejuvenated with non-saline water, transplanted into pots, and intercrossed in the greenhouse to produce seeds for a subsequent cycle. A half-sib family structure was maintained with approximately 2000 plants screened per cycle in each population. BC79S was selected exclusively from BC79, an experimental yellow-flowered Falcata (*M. sativa* subsp. *falcata*) population developed at the USDA ARS Forage and Range Research Laboratory in the late 1970s. CS was derived from the sativa cultivars Malone and Saranac. SII was derived from the sativa cultivars Archer II and Salado. Both CS and SII are purple-flowered alfalfas (*M. sativa* subsp. *sativa*) typical of commercial types. Three lines (half-sib families) were identified from BC79S, three from CS, and four from SII for the extensive evaluation described here. 

### 2.2. Test Sites and Establishment

**Figure 2:**
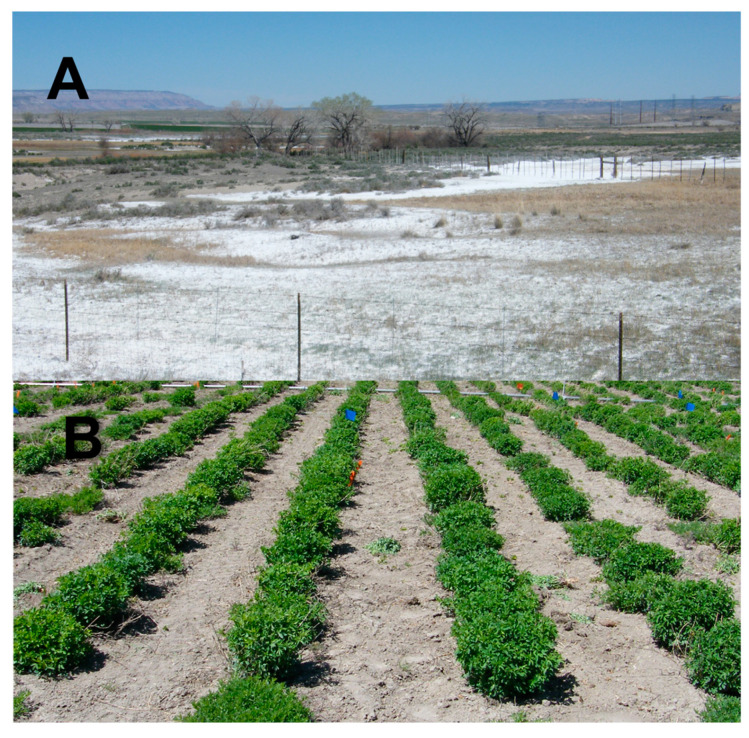
High levels of salt on the soil surface (**A**) due to wicking action located approximately 400 m north-east of the saline study site. Salt-stressed test site (**B**) in early May showing spaced alfalfa plants during early vegetative growth. Soil at this site has been under continuous cultivation for over 50 years.

The field plots were located at the Utah State University Evans Research Farm near Millville, Utah (elevation 1381 m) and at Pacific Corp’s coal-fired electrical power plant located near Castle Dale, Utah (1730 m). Evans was used for the non-stressed test environment, while Castle Dale was used for the salt-stressed test environment. The soil at the Evans Farm is a Nibley silty clay loam (fine, mixed, mesic Aquic Argiustolls) where the annual precipitation averages at 47 cm (1981–2010) [40]. The Evans Farm site is in the Central Great Basin of the western USA and is characterized by hot, dry summers where most of the annual precipitation typically occurs as snowfall. The soil at the Castle Dale site is a Billings silty clay loam (fine–silty, mixed, active, calcareous, mesic Typic Torrifluvents) with an average annual precipitation of 20.5 cm (1981–2010) [40]. The Castle Dale site is in the semi-arid central Great Basin but receives a much lower level of annual precipitation than the Evans Farm site. The Castle Dale site was chosen as a location known to have high salt concentrations, as is illustrated in Figure 2A, showing accumulation in depressions located ~400 m north-east from the plot site (Figure 2B). The Castle Dale site has been under continuous cultivation for over 50 years and was uniform in its soil type and texture within the test area. The soil salinity at a depth of 10 to 15 cm averaged 4.8 ds m^−1^ in late May of 2010 and 4.6 ds m^−1^ in late May of 2011, based on three measurements within each replication. The blocking used and the relatively small replication size of the trial appeared to effectively minimize variations in soil salinity within the replications. Soil EC was measured using a Field Scout EC110 meter (Spectrum Technologies, Plainfield, IL, USA) following the manufacturer’s recommendations. The site was irrigated with water from the powerplant cooling towers, with high concentrations of salt that remained relatively constant throughout, averaging 6.8 ds m^−1^. 

Plant materials were established as transplanted spaced plants in 10-plant plots during the spring of 2009. Using the seeds produced from the last round of greenhouse selection, plants were set up in the greenhouse during the second week of January in Ray Leach Cone-tainers. They were transplanted to the field in the last week of April in Castle Dale and the second week of May at Evans Farm. Four replications were used at Evans Farm while six replications were used at Castle Dale. Six replications were used at the salt-stressed site to mitigate any variability in soil salinity commonly encountered in field tests of this nature [23,37]. Three weeks after transplanting, dead plants were replaced. Plants were established on 33 cm centers in rows, with 1 m spacing between the rows. Due to a limited amount of seeds, we used transplants of the selected lines. Irrigation was applied immediately after planting at the Castle Dale site and as needed thereafter at both locations to ensure establishment. Prior to planting, mono-ammonium phosphate (11N-52P-0K) was applied to both test sites at 90 kg ha^−1^, providing 9.9 and 46.8 kg ha^−1^ of N and P, respectively. This application was made to ensure plant P requirements were met during the study. After the year of establishment, irrigation was applied at Evans Farm approximately one week prior to the first harvest each year and approximately one week after each harvest. Due to the much drier conditions at Castle Dale, irrigation was applied earlier in the spring when the plants had broken dormancy in the third week of April, a week prior to the first harvest, and again approximately one week after each harvest. The plots were hand-weeded and kept weed-free for the duration of the study. 

### 2.3. Data Collection 

Four plants were randomly selected from within each plot and marked with a flag, and all stem length and node number measurements were taken from the same plants for the two years of data collection. Stem length was measured on eight main stems that terminated in an inflorescence originating from the crown of each of the selected plants. The number of nodes was counted on the same eight stems. Nodes were defined as the point at which a leaf or axillary bud originated from the main stem below the inflorescence. Stem length and number of nodes were determined one day prior to harvest for each of the three harvests.

Forage harvests were completed with a Swift Current sickle-bar harvester (Swift Machine & Welding LTD, Swift Current, SK, Canada) to a stubble height of 10 cm. Growth stage was monitored closely with harvests targeted for 15% bloom at each location. The plots at Evans Farm were harvested on 23 June, 27 July, and 23 September in 2010 and on 23 June, 25 July, and 13 September in 2011. The plots at Castle Dale were harvested on 22 June, 20 July, and 14 September in 2010 and on 21 June, 21 July, and 15 September in 2011. At each harvest, a subsample of approximately 400 g was obtained, weighed, and dried at 60 °C to a constant weight. The sample dry weight divided by the wet weight was used to calculate the plot’s total dry weight. The same sample was used for subsequent forage quality analysis and estimating leaf-to-stem ratio (LSR). Leaf-to-stem ratio was estimated at harvests one and two for both locations and years by separating the leaves and stems, weighing them, and dividing the leaf sample weight by the stem sample weight. Samples were then recombined and ground to pass through a 2 mm screen for the determination of CP and NDF levels.

To determine the forage nutritive value in terms of their CP and NDFs, the ground forage samples were scanned with a Near Infrared Reflectance Spectroscopy (NIRS) instrument Foss Rapid Content Analyzer XM-1100 series (Eden Prairie, MN, USA). System software was used to select ~10 percent of the samples to calibrate an existing in-house equation for alfalfa. These samples were not used for equation development. The samples used for calibration were analyzed for N content using an LECO CHN-2000 series Elemental Analyzer (LECO Corp, St. Joseph, MO, USA) and multiplied by 6.25 to obtain the CP values. The ANKOM 2000 Fiber Analyzer (ANKOM Technology Corporation, Macedon, NY, USA) was used to estimate NDF levels, employing the procedures of Goering and Van Soest [41] as modified in the ANKOM procedures [42]. Validations of the equations were determined from a different subset of samples for CP and NDFs. The r^2^ values (standard error of prediction) were 0.98 (0.68) for CP, and 0.95 (1.96) for NDFs. The CP and NDF forage nutritive data were completed on harvests one and two at each location and in each year.

### 2.4. Design and Analysis

The study was arranged as a randomized complete block design with four replications at the Evans Farm site (non-stressed) and six replications at the Castle Dale site (salt-stressed). Data were analyzed using the General Linear Model procedure (SAS Institute Inc., Cary, NC, USA). Entry and location were considered fixed effects, and replications and years were considered random effects. Parents of the individual populations were combined for the analysis. The salt-stressed vs. non-stressed locations were significantly different (*p* < 0.001) for all traits and are presented separately. In the non-stressed test, Spearman’s rank correlation coefficients were all between r = 0.74 and r = 0.91 across both harvests and years with no major changes in rank for leaf-to-stem ratio, stem length, and node number, thus they were combined. Similarly, in the salt-stressed test, Spearman’s rank correlation coefficients were between r = 0.81 and r = 0.98 across both harvests and years with no major rank changes for leaf-to-stem ratio, stem length, and node number, thus they were also combined. Entry x harvest was significant for forage mass, thus harvests are presented separately. The primary focus was on the relative differences between the selected lines and their parents under salt-stressed versus non-stressed conditions. Fisher’s protected LSD at *p* = 0.05 was applied for the mean separation.

## 3. Results

### 3.1. Forage Mass 

**Figure 3:**
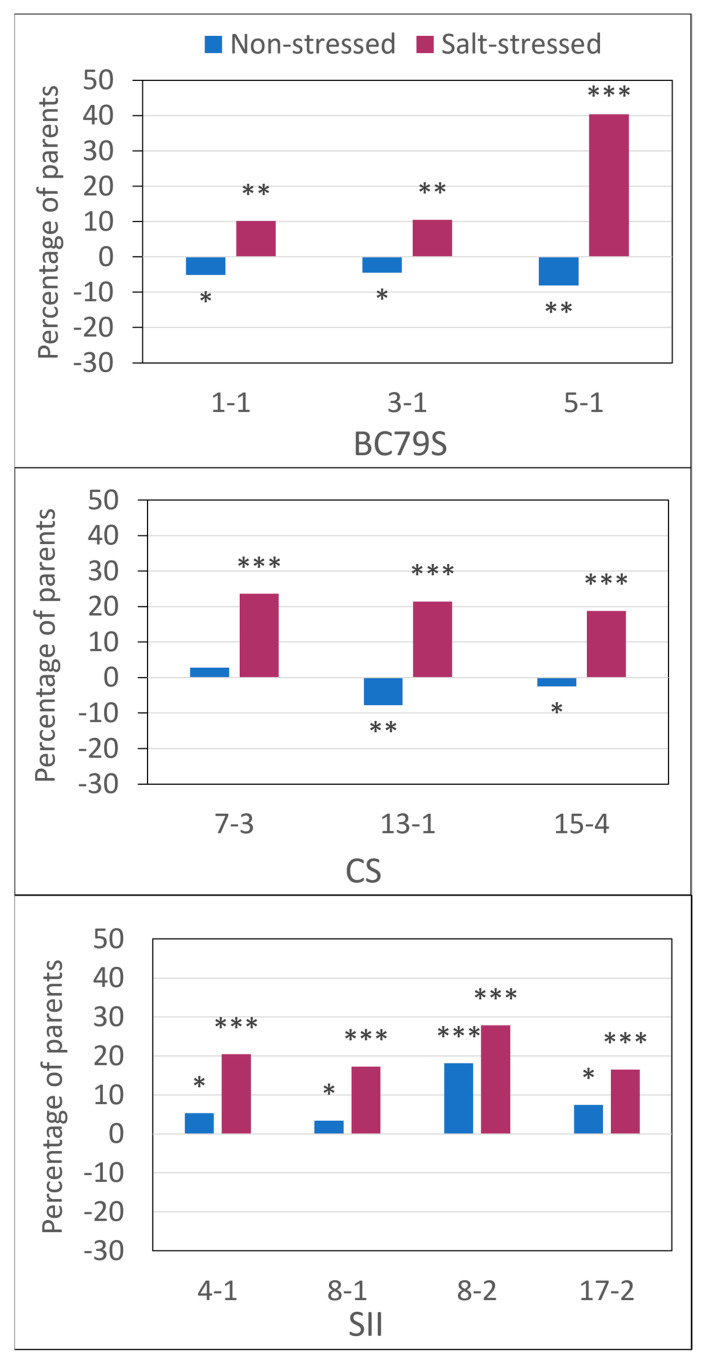
Total seasonal forage mass of lines from three populations selected for their tolerance to saline conditions as a percent of their parents’, when grown in a non-stressed (blue bars) versus salt-stressed (maroon bars) environment. Lines with bars that fall below zero produced a forage mass less than their parental mean while bars above zero produced a forage mass greater than their parental mean. *, ** and *** indicate significant differences from the parental mean at *p* = 0.05, 0.01, 0.001.

The overall forage mass in the non-stressed test was significantly (*p* = 0.001) greater than that of the salt-stressed test. Across all entries, the overall total seasonal production was 9562 versus 5783 kg ha^−1^ in the non-stressed versus salt-stressed tests, respectively, showing nearly a 40% production advantage for the non-salt-stressed site (Table 1). The forage mass advantage in the non-stress test was universally observed in all selected lines and their parents; even so, there were differences within lines of the different populations. The total seasonal forage mass of the selected lines of BC79S and CS in the non-stressed site averaged 4% lower than their respective parents (Figure 3). However, this was not consistent across harvests, as in the BC79S harvests one and two, the lines were either not different or trended lower, while in harvest three all lines were lower than the parent (Table 1). In each individual harvest in the non-stressed test, CS 7-3 was not different to its parental mean but was significantly greater in total seasonal production (Table 1). In SII, the lines had either greater forage mass or were not different than the parental mean in the individual harvests, while the total seasonal forage mass of all lines was greater than the parental mean in the non-stressed test.

**Figure 4:**
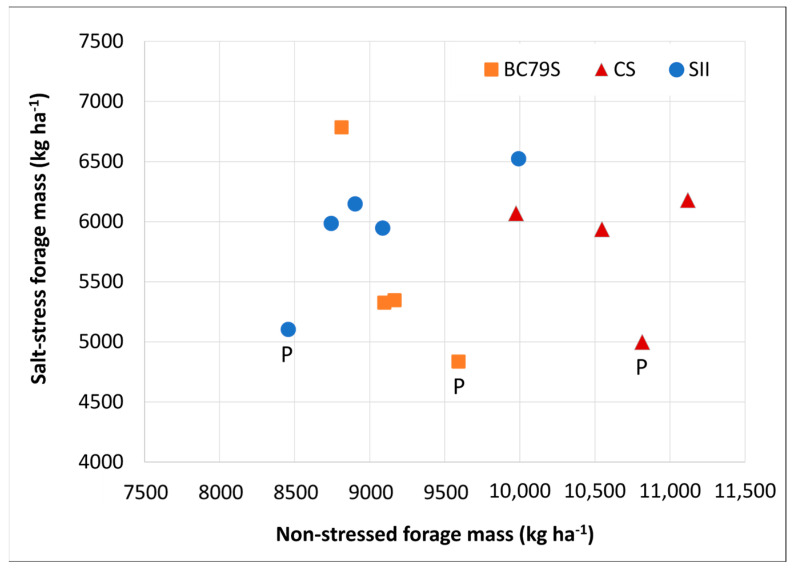
Spatial relationship between the non-salt-stressed and salt-stressed field conditions for the total seasonal forage mass of alfalfa lines from three populations (BC79S, CS, and SII) selected for salt tolerance. Data points with a P denotes parental mean of their respective population.

While forage mass in the salt-stressed environment was lower overall, the selected lines averaged a 20% greater overall production than that of their respective parental means in each of the three populations (Table 1, Figure 3). The production advantage of the selected lines over their parents was relatively consistent in the CS and SII lines, while in the BC79S line, 5-1 had 40% greater overall production than its parent with the other two lines each showing a 10% production advantage (Figure 3). In both the BC79S and CS lines, the difference between them and their parents was the least in harvest one (Table 1). Among the BC79S lines, only 5-1 had a greater forage mass at harvest one; this changed, however, increasing to an overall 23 and 52% advantage by the selected lines in harvests two and three, respectively. In the SII materials, the yield of the lines was consistently 20% above the parental mean, regardless of harvest. For both CS and SII, the yield of all lines was greater than the parental mean at all harvests; whereas, in harvest one, the BC79S line 1-1 was not different than its parent and 3-1 was lower, but both lines were greater at harvests two and three (Table 1, Figure 3). The relationship of the seasonal forage mass production values between the non- and salt-stressed tests (Figure 4) illustrates how each of the lines from the populations behaved differently. The forage mass values of the SII lines were each greater than their parental means in both tests compared to BC79S, with one line excelling in the salt stress test and nothing otherwise noteworthy. In contrast, the forage mass of the CS lines was good in the non-stressed test but very stable, and showed no change in the salt-stressed test.

### 3.2. Stem Length, Leaf-to-Stem Ratio (LSR), and Number of Nodes

Stem length averaged 61 cm in the non-salt-stressed test and was significantly lower (*p* = 0.001) in the salt-stressed test, at 43 cm (Table 2). An overall reduction in stem length between the two tests was universal across the populations, but even so there were differences among them. In the non-stressed test, the BC79S parent had on average a 10 cm greater stem length than the selected lines, whereas in the salt-stressed test, it averaged at a just under 4 cm shorter stem length. BC79S lines 1-1 and 5-1 were the only lines among any of the populations that exceeded their parent’s stem length in the salt-stressed test. The SII population was somewhat similar, in that the stem length of the parental mean in the non-stressed test was greater than two lines and no different than the remaining two lines, whereas in the salt stress test, the selected lines were no different than the parental mean. Contrastingly, in the CS population, all selected lines had a greater stem length in the non-stressed test and were no different in the salt-stressed test. However, the relative reduction in stem length of the selected lines versus the parental mean was much greater in the CS lines.

The LSR values between the non-stressed and salt-stressed tests were also different (*p* = 0.001), showing an overall increase from 0.95 to 1.26. In the non-stressed test, most LSR values of the selected lines and those of their parents did not differ in the BC79S and CS populations, though BC79S 1-1 was an exception, being greater than its parent (Table 2). In SII, three of the four lines had a leaf-to-stem ratio lower than that of the parental mean and one that was not different in the non-stressed test. In both the CS and SII, populations with sativa backgrounds, the LSR tended to be below 1.0 in the non-stressed test, with one exception in the SII. In contrast, in the salt-stressed test, the LSR of the CS and SII material with a sativa background, including that of their parents, tended to be greater than 1.0. While the ratio increased to above 1.0 in both CS and SII, these lines tended not to differ from their respective parental mean in the salt-stressed test. In the BC79S lines and parents, the LSR values all increased to above 1.5 in the salt-stressed test. While the BC79S lines were not different or greater than their parental mean in the non-salt-stressed test, under salt stress, all BC79S lines were significantly less than their parental means.

Similar to the stem length and LSR, the node number decreased significantly (*p* = 0.001) from the non-stressed to the salt-stressed test, going from an average of 12.7 to 9.3, respectively: a 36% reduction (Table 2). The node numbers of the parents and lines in the non-salt-stressed test showed no differences between the SII parents and lines, while two CS lines showed a greater number of nodes than their parents. Conversely, BC79S lines 1-1 and 3-1 had fewer nodes than their parents in the non-stressed test. In the salt-stressed test this was reversed, where the same two BC79S lines, 1-1 and 3-1, had more nodes than their parent. Except for the 8-1 SII line, there were no differences between lines and their respective parents for either the CS or SII in the salt-stressed test.

### 3.3. Forage Nutritive Value

The overall CP content averaged near 200 g kg^−1^ in the non-stressed test and was significantly higher, at 231 g kg^−1^, in the salt-stressed test (Table 3). This increase in CP from the non-stressed to the salt-stressed test was similar for all selected lines within each population and their respective parents. In the non-stressed environment, SII lines 8-2 and 17-2 were lower than their parental means, but no other lines differed from their respective parental mean. In the salt-stressed test, CS 7-3 and SII 8-2 were lower than their respective parental mean, each by less than 2%. 

Similar to the CP, the NDF was slightly higher, or less desirable, in the non-stressed test, at 345 g kg^−1^, than in the salt-stressed test, at 329 g kg^−1^ (Table 3). While this difference between the test environments is significant (*p* = 0.01), it is not large. Overall, the differences between the selected lines and their respective parental means were few. Within the non-stressed site, SII 8-1 was lower than its parental mean, with no other differences between the lines and their parents. In the salt-stressed test, two BC79S lines and one CS line showed greater NDF levels than their respective parents.

## 4. Discussion

The overall decrease reported here in forage mass production at the salt-stressed site supports the assertation put forth by Johnson et al. [8] and Rawlins [9] that a decrease in alfalfa yield can be expected when soil salinity is above 2.0 dS m^−1^ (~22 mM NaCl). However, the forage mass reduction observed in this study was greater than 7.0 to 7.3% for each dS m^−1^ (~11 mM NaCl) increase above a baseline of 2. If this formula is applied based on a soil salinity of 4.8 then the reduction would be about 20%. If the formula is applied based on the salinity of the irrigation water, the reduction in forage mass would be around 35%. However, both of these values are much lower than those actually observed. While this study was not meant to delineate the expected reduction per unit increase in salinity, it is reasonable to state that our observations support a reduction in production as put forth by Johnson et al. [8] and Rawlins [9], with the qualifier that they may have underestimated the decrease in forage mass associated with incremental increases in salt concentration. Even so, the high EC of the soil combined with the high EC of the irrigation water assure that the test was indeed salt-stressed.

Comparing the forage mass production of the BC79S and CS lines at the two test sites, with their parent values used as the baseline, indicates the presence of a genotype via the environmental interactions with the rank changes. As such, lower forage mass in the non-stressed tests of the BC79S and CS lines suggests the possible occurrence of negative changes associated with selection. However, when the SII lines were examined, there were no rank changes between the two tests of lines when compared with their parental mean, with each test having a total season forage mass production greater than their parental mean. Simmonds [43] suggested that, ideally, selection for a trait would reduce any genotype by environmental effect to zero. However, when dealing with an abiotic stress such as salinity and comparing a non-salt-stressed site to a salt-stressed site, it is unlikely that reductions in productivity will be avoided. A more reasonable approach than having zero genotype by environmental effects of zero across the environments would be to identify the lines that produce well in both locations. If this was the approach followed, then the CS 7-3 and SII 8-2 lines would both qualify, having greater production values than their parental means in both tests. Alternatively, when the target environment is severely stressed, then it may be more advantageous to utilize a line like BC79S 5-1, with greater performance at all harvests and overall production under stress but lower productivity in a non-stressed environment.

Munns and Tester [7] assert that salt stress has a universal effect of reducing the biomass and growth rate of both shoots and leaves. Our aim was to document changes in stem length, LSR, and number of nodes per stem that might be associated with selection for salt tolerance. With regard to stem length, the overall reduction was universal from the stressed to the non-stressed test, supporting the reports from both Campanelli et al. [38] and Al-Ashkar et al. [39] of similar reductions in alfalfa plant height and wheat shoot length, respectively. However, the differences among the lines of each population group are considerable. The BC79S lines showed a lower reduction in height from non- to salt-stressed tests relative to their parent, while the CS lines showed a greater non-stressed height, which is likely a function of their different genetic backgrounds. Even so, there was not a universal change in stem length associated with selection. However, any height reduction would relate to corresponding changes in LSR. The universal increase in LSR under salt stress relative to the non-stressed test demonstrates that stem mass was diminished more than leaf mass (Table 2). We saw an overall decrease of about 22 cm (30%) from the non-stressed to salt-stressed tests, closely corresponding to an overall increase in LSR of 32% from the non-salt- to the salt-stressed tests. When looking for differences between the selected lines and unselected parents in terms of LSR, it does appear that the selected lines from BC79S showed less change than their parent between the tests but a greater change than for either of the other population groups.

No specific examples are found in the literature referencing the impact of salt stress on the number of nodes. However, the reduction observed here does follow the assertion from Munns and Tester [7] of a universal reduction in growth under salt stress. In evaluating the effect of selection, the comparison of lines with their parents in the non- and salt-stressed tests suggests that the number of nodes was not affected in the CS and SII populations. In BC79S, the lines went from averaging less than their parent in the non-stressed test to more under salt stress, indicating that selection made those lines less susceptible to change in terms of their number of nodes.

We found no evidence that the two measures of forage nutritive value, CP and NDF, were impacted by the selection for salt tolerance per se. Hu et al. [44] does report a parabolic relationship between CP and salinity in alfalfa, by which CP increased until approximately an EC of 3.5 ds m^−1^ was reached. However, they were evaluating the overall impact of salinity on alfalfa in general, and in that context, our results tend to support those of Hu et al. [44]. In the context of a comparison between non- and salt-stressed tests, the higher CP of the salt-stressed test likely reflects the increase in LSR as leaf material is known to have much higher level of CP than stem material. The same would also be true for the slightly lower (more desirable) NDF in the salt-stressed test, where the NDF of the leaf material would be much lower than that of the stems.

In evaluating the significance of this report, the greenhouse selection protocol of Peel et al. [37] used to develop the material, which presumably had limitations as it selected for plant survival, was effective for improving the production of forage mass per se in a field test. The purpose of this protocol’s development was to eliminate the challenges associated with selection under field conditions where salt conditions are variable [23,24]. It is therefore encouraging that we found selected lines from each of the populations whose forage mass exceeded that of their parents in a non-stressed test. Furthermore, we found a universal increase in the forage mass of selected lines under salt stress. This, combined with the previous reports of Anower et al. [12,19] that showed a greater accumulation of chlorophyll content, improved relative water content, and lower sodium accumulation, indicates that the greenhouse selection protocol is a successful breeding tool based on the criteria set forth by Ceccarelli [36].

## Figures and Tables

**Table 1:** Forage mass under non-stressed and salt-stressed test environments of alfalfa lines selected for salt tolerance in comparison with their unselected parental means, in 2010 and 2011.

Population/Line	Harvest 1		Harvest 2		Harvest 3		Yearly Production	
	Kg ha^−1^							
BC79S	Non-Stressed							
1-1	4144	a	2497	b	2456	b	9098	b
3-1	4230	a	2646	ab	2289	c	9166	b
5-1	3737	b	2843	a	2235	c	8814	c
Parental Mean	4165	a	2791	a	2636	a	9592	a
CS								
7-3	3693	ab	3683	a	3744	a	11,119	a
13-1	3480	b	3255	c	3240	b	9975	d
15-4	3583	ab	3415	bc	3550	a	10,547	c
Parental Mean	3732	a	3475	b	3608	a	10,816	b
SII								
4-1	3732	a	2625	c	2547	b	8904	bc
8-1	3335	bc	2938	bc	2471	b	8744	c
8-2	3710	a	3335	a	2946	a	9992	a
17-2	3462	b	3144	ab	2482	b	9087	b
Parental Mean	3147	c	2848	c	2463	b	8457	d
BC79S	Salt-Stressed							
1-1	2142	b	1602	b	1581	c	5325	b
3-1	2057	b	1564	bc	1723	b	5344	b
5-1	2451	a	2102	a	2231	a	6784	a
Parental Mean	2193	b	1427	c	1215	d	4835	c
CS								
7-3	1960	b	1910	a	2307	a	6178	a
13-1	2138	a	1912	a	2019	b	6069	ab
15-4	1983	b	1891	a	2063	b	5937	b
Parental Mean	1787	c	1516	b	1695	c	4998	c
SII								
4-1	2307	ab	1850	ab	1990	b	6147	b
8-1	2133	bc	1778	b	2074	b	5986	c
8-2	2118	bc	1991	a	2414	a	6524	a
17-2	1996	c	1851	ab	2101	b	5947	c
Parental Mean	1768	d	1549	c	1787	c	5103	d

^a–c^ Means within a column under a population heading with different superscripts are significantly different (*p* = 0.05).

**Table 2:** Stem length, leaf-to-stem ratio (LSR), and number of nodes per stem of alfalfa lines selected for increased salt tolerance and their parental means under non-stressed and salt-stressed test environments, in 2010 and 2011.

Population	Stem Length				LSR				Nodes per Stem			
	Non-Stressed		Salt-Stressed		Non-Stressed		Salt-Stressed		Non-Stressed		Salt-Stressed	
	cm								Count			
BC79S												
1-1	46.2	c	35.8	a	1.08	a	1.53	b	11.3	b	9.6	a
3-1	50.0	c	31.8	bc	1.04	ab	1.59	b	11.8	ab	9.5	a
5-1	56.3	ab	35.2	ab	1.01	ab	1.55	b	12.4	a	8.7	ab
Parental Mean	60.3	a	30.8	c	0.94	b	1.81	a	12.5	a	8.4	b
CS												
7-3	77.7	a	47.8	a	0.79	a	1.17	a	13.9	a	9.6	a
13-1	68.3	b	46.6	a	0.88	a	1.12	a	13.4	ab	10.0	a
15-4	71.6	b	46.2	a	0.92	a	1.06	a	14.1	a	9.7	a
Parental Mean	63.0	c	45.9	a	0.81	a	1.12	a	12.6	b	9.5	a
SII												
4-1	63.6	ab	50.2	a	0.98	bc	0.95	b	12.7	a	9.3	a
8-1	53.9	c	45.6	b	1.04	ab	1.14	a	12.1	a	8.4	b
8-2	64.6	a	49.7	a	0.89	c	1.08	a	12.4	a	9.7	a
17-2	60.5	b	46.2	ab	0.90	c	1.12	a	12.9	a	9.3	a
Parental Mean	65.0	a	46.8	ab	1.13	a	1.14	a	12.9	a	9.6	a

^a–c^ Means within a column under a population heading with different superscripts are significantly different (*p* = 0.05).

**Table 3:** Forage nutritive value of alfalfa lines selected for increased salt tolerance and their parental means in terms of their crude protein and neutral detergent fibers under non-stressed and salt-stressed test environments, in 2010 and 2011.

Population	Crude Protein				Neutral Detergent Fibers			
	Non-Stressed		Salt-Stressed		Non-Stressed		Salt-Stressed	
	g kg^−1^							
BC79S								
1-1	201	b	237	a	346	a	318	b
3-1	211	a	247	a	332	a	305	c
5-1	205	ab	234	b	330	a	335	a
Parental Mean	205	ab	243	ab	345	a	305	c
CS								
7-3	191	a	214	a	361	a	353	a
13-1	198	a	230	a	348	a	332	b
15-4	192	a	230	a	362	a	331	b
Parental Mean	195	a	228	a	362	a	334	b
SII								
4-1	209	a	229	a	330	bc	337	ab
8-1	203	ab	231	a	325	c	328	b
8-2	193	c	219	b	343	ab	343	a
17-2	190	c	228	ab	358	a	333	ab
Parental Mean	201	b	232	a	345	ab	331	ab

^a–c^ Means within a column under a population heading with different superscripts are significantly different (*p* = 0.05).

## Data Availability

The data presented in this study are openly available in FigShare at 10.6084/m9.figshare.24199731.
